# Seroprevalence of *Toxoplasma gondii*, *Neospora caninum* and *Encephalitozoon cuniculi* in Red Foxes (*Vulpes vulpes*) from Italy

**DOI:** 10.3390/pathogens14111175

**Published:** 2025-11-18

**Authors:** Leonardo Brustenga, Stefano Scarcelli, Giulia Rigamonti, Iolanda Moretta, Manuela Diaferia, Giulia Morganti, Nicoletta D’Avino, Marco Gobbi, Alice Ranucci, Giovanni Sgroi, Fabrizio Passamonti, Fabrizia Veronesi

**Affiliations:** 1 Department of Veterinary Medicine, University of Perugia, Via San Costanzo 4, 06126 Perugia, Italy; giulia.rigamonti@dottorandi.unipg.it (G.R.); iolanda.moretta@unipg.it (I.M.); manuela.diaferia@unipg.it (M.D.); giulia.morganti@unipg.it (G.M.); fabrizio.passamonti@unipg.it (F.P.); fabrizia.veronesi@unipg.it (F.V.); 2 WildUmbia, Wildlife Rescue Center, Loc Candeleto, 06026 Pietralunga, Italy; 3 SELVA-VET, Veterinary Research Center on Wildlife, University of Perugia, Via San Costanzo 4, 06126 Perugia, Italy; 4 Department of Veterinary Medicine and Animal Productions, University of Naples Federico II, Via Delpino 1, 80137 Naples, Italy; stefano.scarcelli@unina.it; 5 Experimental Zooprophylactic Institute of Umbria and Marche, Via Gaetano Salvemini 1, 06126 Perugia, Italy; n.davino@izsum.it (N.D.); m.gobbi@izsum.it (M.G.); a.ranucci@izsum.it (A.R.); 6 Experimental Zooprophylactic Institute of Southern Italy, Via Salute 2, 80055 Naples, Italy; giovanni.sgroi@izsmportici.it; 7 Department of Sciences and Technologies (DST), University of Sannio, 82100 Benevento, Italy

**Keywords:** *Toxoplasma gondii*, *Neospora caninum*, *Encephalitozoon cuniculi*, *Vulpes vulpes*, IFAT, Italy

## Abstract

The ecological role and overlap with urban environments make wild carnivores useful epidemiological sentinels for several pathogens. The present study aimed to investigate the seroprevalence of *Toxoplasma gondii*, *Neospora caninum* and *Encephalitozoon cuniculi* in red foxes (*Vulpes vulpes*) from Central and Southern Italy. Sera from 120 foxes were analyzed using IFAT with a 1:20 cut-off value. Overall, seropositivity was highest for *T. gondii* (68.5%), followed by *E. cuniculi* (15.0%) and *N. caninum* (3.3%). Multivariable logistic regression models with stepwise selection identified age class and location as significant predictor factors for *T. gondii* exposure, with adults and red foxes from Southern Italy showing higher levels of prevalence. No significant associations with epidemiological risk factors were detected for *E. cuniculi* or *N. caninum*. Co-infections were detected in 15% of red foxes with a statistically significant positive association between *T. gondii* and *E. cuniculi*. These findings highlight that red foxes, being scavengers, are particularly exposed to food-borne pathogens, especially to *T. gondii*, and prove once again that they are reliable epidemiological sentinels for parasites that circulate at the wild–domestic interface.

## 1. Introduction

Wildlife plays an important role in the lifecycle of many pathogens including parasites [1]. For some parasites, the sylvatic cycle represents the only route of diffusion (i.e., *Echinococcus multilocularis*); on the contrary, several other parasites are able to circulate through both the sylvatic and domestic environment, exploiting the most diverse routes of transmission [2]. The red fox (*Vulpes vulpes*) is the most widely distributed wild carnivore worldwide, occurring across Europe, Asia, North America and Australia, where it thrives in wild, rural and, increasingly, in urban environments [3]. The remarkable ecological plasticity confers on red foxes a highly opportunistic feeding strategy, from active predation to the consumption of vegetable matter and scavenging on carcasses and human garbage. This broad dietary spectrum, combined with the ever more frequent interactions with anthropogenic food sources, makes the red fox an excellent sentinel species for monitoring zoonotic and food-borne pathogens, like *Toxoplasma gondii* [4].

*Toxoplasma gondii*, *Neospora caninum* and *Encephalitozoon cuniculi* are important pathogens of veterinary concern that share a specific tropism for the brain of many vertebrate species [2]. While *N. caninum* is not able to infect humans, *T. gondii* and *E. cuniculi* share a zoonotic potential and are particularly harmful to immunocompromised patients [5,6].

*Toxoplasma gondii* and *N. caninum* are both obligate intracellular cyst-forming protozoa (Phylum: Apicomplexa) with an indirect lifecycle that uses carnivores as definitive hosts, felids are the definitive hosts for *T. gondii*, whilst canids are the definitive hosts for *N. caninum* [7]. A wide array of warm-blooded vertebrates serve as intermediate hosts that can acquire the infection by accidentally ingesting oocysts that are shed in the environment by the definitive hosts, by consuming the contaminated flesh of other hosts or by vertical transmission from mothers to offspring [8]. Although *T. gondii* and *N. caninum* are responsible for economic losses in livestock production, being aborting agents for sheep and cattle, respectively [9,10], infections are usually subclinical in wild species [6,11,12]. Microsporidia are also obligate intracellular parasites but are conversely characterized by a direct lifecycle that can be sustained by a very diverse host range that comprises both vertebrates and invertebrates [5]. Within the genus *Encephalitozoon*, *E. cuniculi* and *E. intestinalis* are the two species that were reported to cause disease in both humans and other domestic and wild mammals [13,14]. While the zoonotic potential of *E. intestinalis* is yet to be clarified [14], the role of *E. cuniculi* is much clearer and more studied [8], with several reports of severe neurologic signs caused by encephalitozoonosis in both canids, like dogs (*Canis familiaris*) or Arctic foxes (*Vulpes lagopus*), lagomorphs and rodents, that are considered as natural reservoirs sustaining the transmission cycle in the wild [8].

The present study aimed to investigate the seroprevalence of the three neurotropic pathogens in red foxes from Central and Southern Italy, with the aim of assessing their circulation and potential epidemiological role.

## 2. Materials and Methods

### 2.1. Sampling Area and Sample Collection

Samples from 120 red foxes were collected from Umbria, Marche and Campania regions from November 2023 to June 2025. Umbria and Marche will hereafter be categorized as Central Italy, whereas Campania as Southern Italy. Peripheral blood was drawn from live foxes that were rescued by the WildUmbria wildlife rescue center and admitted to the Veterinary Teaching Hospital of Perugia University (Central Italy), while clotted blood was harvested upon necropsy from the heart of dead foxes brought to the Experimental Zooprophylactic Institutes of Umbria and Marche (Central Italy) and of Campania (Southern Italy). Blood clots were collected in falcon tubes, broken by pipetting with sterile pasteur pipettes and then centrifuged at 4000× *g* for 10 min to pellet cellular debris and then recover the serum. Blood from live foxes was drawn at the Veterinary Teaching Hospital by authorized veterinarians. All venipunctures were performed as part of routine serological screening to guide the clinical treatment of the animals and to establish therapeutic protocols. Blood samples and broken blood clots were centrifuged to obtain serum, and sera were stored at −20 °C until processing. Sex was visually determined, whereas age class (juvenile or adult) was inferred by examining incisor teeth wear [15].

### 2.2. Serological Analysis

The presence of antibodies (IgG) against *T. gondii, N. caninum* and *E. cuniculi* was investigated by using an immuno-fluorescent antibody test (IFAT) performed using three commercial IFAT assays: MegaFLUO^®^ TOXOPLASMA g., MegaFLUO^®^ NEOSPORA c. and MegaFLUO^®^ ENCEPHALITOZOON c. (Megacor Diagnostik, Hoerbranz, Austria). Two-fold serial dilutions of sera were performed, antigen-coated slides were incubated for 30 min at 37 °C with 20 µL of serum, washed in PBS and then incubated for 30 min at 37 °C with a drop of fluorescein-marked FITC anti-dog IgG conjugate (Megacor Diagnostik, Hoerbranz, Austria) to bind the antigen–antibody complex. Slides were evaluated with an Olympus BX51 fluorescence microscope at 400× magnification and samples were considered as positive for all the three parasites when they exhibited fluorescence in dilutions equal or higher to 1:20 [16,17]. End-point titers, i.e., the last dilution to exhibit fluorescence, were determined for all the positive samples. All reactions were carried out along the supplied positive and negative controls (Megacor Diagnostik, Hoerbranz, Austria).

### 2.3. Statistical Analysis

The serological results were analyzed to determine the prevalence and distribution of *T. gondii*, *N. caninum* and *E. cuniculi* in red foxes from Central and Southern Italy. Total prevalence for each pathogen as well as stratified prevalence according to sex, age class and location were calculated along with the relative 95% confidence intervals (CIs). Multivariable logistic regression models with stepwise selection were used to identify the epidemiological factors associated with seropositivity for each of the pathogens, using sex, age class and location as possible predictor factors of infection [18]. Inferential statistical analyses were carried out to rule out statistically significant differences among the regions of Central Italy (Umbria and Marche), and the status (alive and deceased) of the sampled animals in the prevalences detected for the three pathogens. Differences in serological titers among levels of the epidemiological factors were assessed with the non-parametric Mann–Whitney test [19]. An UpSet plot [20] was used to visualize co-infections, while pairwise associations between pathogens were tested using Fisher’s exact test, calculating odds ratios (OR) and assessing statistical significance at *p* values < 0.05. All statistical analyses were carried out in R v4.3.0.

## 3. Results

Out of 120 foxes, 60 (50%) foxes from Central Italy were alive at the time of sampling and the remaining 60 (50%) were carcasses from Central (21, 35%) and Southern Italy (39, 65%). Overall, 81 (67.5%) animals were sampled in Central Italy, whereas 39 (32.5%) animals were sampled in Southern Italy. Sex and age class were successfully determined for all the animals: 70 (58.3%) were males and 50 (41.7%) were females whereas 58 (48.3%) were adults and 62 (51.7%) were juveniles.

The total seroprevalence was highest for *T. gondii* (79/120, 65.8% 95% CI 57–73.7) with titers ranging from 1:20 to ≥1:2800, followed by *E. cuniculi* (18/120, 15.0% 95% CI: 9.7–22.5) with titers ranging from 1:20 to 1:320 and lastly by *N. caninum* (4/120, 3.3% 95% CI: 1.3–8.3) with titers ranging from 1:20 to 1:80. Seroprevalence stratified by epidemiological factors (sex, age class and location) highlighted that seroprevalence for *T. gondii* was higher in adults (46/58, 79.3% 95% CI: 67.2–87.7) compared to juveniles (33/62, 53.2% 95% CI: 1.3–8.3) and in foxes from Southern Italy (31/39, 79.5% 95% CI: 64.5–89.2) compared to foxes from Central Italy (48/81, 59.3% 95% CI: 48.4–69.3). Sex did not appear to significantly influence *T. gondii* seropositivity with females being slightly more seropositive than males. No clear patterns emerged for *E. cuniculi*, except that adults (12/58, 20.7% 95% CI: 12.3–32.8) were more seropositive compared to juveniles (6/62, 9.7% 95% CI: 4.5–19.5), or for *N. caninum*. All the stratified seroprevalences are shown in Table 1.

The multivariable logistic regression with stepwise selection of epidemiological factors used as predictors of infection revealed that age class and location were significant predictors for *T. gondii* infection, as reported in Table 2.

None of the factors were retained in the final models for either *E. cuniculi* or *N. caninum*, indicating no significant association with sex, age class and location.

No statistically significant differences (*p* > 0.05) emerged from Fisher’s exact test for any of the pathogens (*T. gondii*: *p* = 0.15, *N. caninum*: *p* = 0.083, *E. cuniculi*: *p* = 1) between Umbria and Marche. The χ^2^ test showed a statistically significant difference between the seroprevalence in deceased vs. alive foxes only for *T. gondii* (*p* = 0.028) with deceased foxes having higher seroprevalence rates compared to live ones.

A comparison of serological titers with the Mann–Whitney test indicated that juveniles had significantly lower (*p* = 0.019) titers of anti-*T. gondii* IgG compared to adults, while no significant differences were detected for sex and location. The test showed no statistically significant differences (*p* > 0.05) for both *E. cuniculi* and *N. caninum* antibody titers across all the tested epidemiological factors.

**Figure 1:**
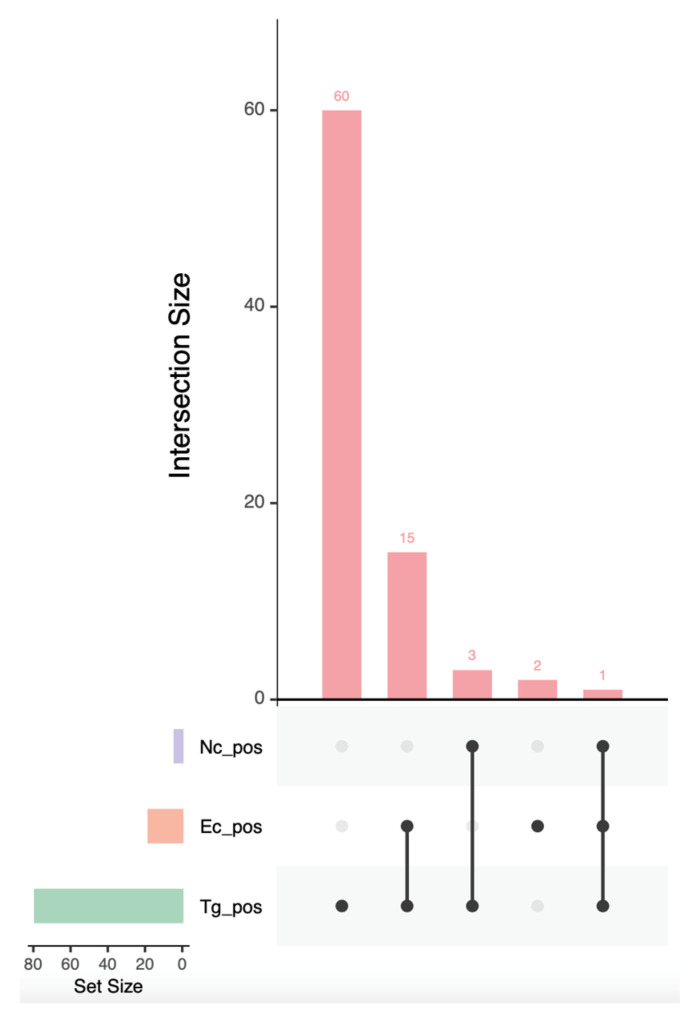
UpSet plot showing the extent of coinfections for *Toxoplsma gondii* (green), *Encephalitozoon cuniculi* (orange) and *Neospora caninum* (purple) in red foxes from Central and Southern Italy. The horizontal bar plot indicates the overall number of red foxes positive to each pathogen. The vertical bar plot indicates the number of foxes that were, respectively, positive only to *T. gondii*; positive to *T. gondii* and *E. cuniculi*; positive to *T. gondii* and *N. caninum*; positive only to *E. cuniculi*; and positive to all three of the pathogens.

The UpSet plot (Figure 1) allowed for us to elucidate the extent of coinfections, showing that 18 foxes (15%, 18/120) were simultaneously positive to two pathogens, with the most frequently observed coinfection (83.3%, 15/18) being caused by *T. gondii* and *E. cuniculi*, while just one fox (5.5%, 1/18) was simultaneously positive to all three pathogens.

The results of Fisher’s exact test revealed a significant association between *T. gondii* and *E. cuniculi* (*OR* = 4.90 95% CI: 1.06–46.25, *p* = 0.03), while no significant associations were detected between *T. gondii* and *N. caninum* (*p* = 0.298) or between *E. cuniculi* and *N. caninum* (*p* = 0.483).

## 4. Discussion

A high seroprevalence to *T. gondii* (65.8%) was observed in foxes from Central and Southern Italy, with animals from the Southern region being significantly more prevalent than those from Central Italy. According to a recent systematic review, the global pooled prevalence of *T. gondii* in red foxes is estimated to be around 46.8% [21], even though studies from both Italy [16] and Europe report higher prevalence values that align with the seroprevalence detected in this study. Similarly, high seroprevalence values for *T. gondii* were detected in foxes from Slovakia (62.7%, 190/303) [22], Spain (64.7%, 66/102) [23], Hungary (67.7%, 228/337) [24], Poland (77.5%, 87/102) [25] and the Czech Republic (100%, 80/80) [26]. Only one study, carried out on 191 foxes from Central Italy, tested for anti-*T. gondii* antibodies, reporting a seroprevalence of 53.4% [16]. On the other hand, molecular prevalence was ascertained across the Italian peninsula with *T. gondii* DNA being detected in the tissue of foxes from Northern (20.2%, 19/94) [27], Central (8.8%, 9/102) [16] and Southern Italy (23.9%, 17/71) [28]. Unsurprisingly, the prevalence values obtained from molecular testing are lower than the seroprevalence estimate, a scenario to be expected given the stochastic possibility of not including parasitic elements while sampling, as parasitic cysts are not uniformly distributed in host tissues [29]. Furthermore, a comparison between serological techniques has shown moderate degrees of agreement between different techniques (DAT and ELISA) but no agreement between serological and molecular tests, demonstrating that serological methods are much more reliable in detecting exposure and prevalence at the population level compared to molecular methods [25].

A significant difference in *T. gondii* seroprevalence was detected between foxes from Southern Italy (79.5%) and Central Italy (59.3%). Although location emerged as a statistically significant factor for *T. gondii* infection, this result might have been biased by the uneven sampling effort, with only 39 foxes collected in Southern Italy compared to the 81 sampled in Central Italy. Nevertheless, this result might be supported by two main factors: (*i*) Southern Italy is characterized by warmer temperature and, in several coastal areas, by higher humidity and rainfall, conditions that favor the survival and sporulation of *T. gondii* oocysts in the environment; (*ii*) Central Italy is more dominated by hills and mountains with a cooler climate that is comparatively less suitable for oocyst persistence. The density of the definitive host, the domestic cat (*Felis catus*), is also a factor that cannot be overlooked; cats are not homogenously distributed across the country, according to 2008 data from the Italian Ministry of Health showing a presumptive regional census of stray cats (https://www.informazioneeditoria.gov.it/media/2505/scheda_dati_regionali_randagismo.pdf accessed on 29 September 2025); Umbria and Marche together were estimated to host approximately 25,042 stray cats, whereas Campania alone accounted for an estimated 70,003 stray cats. In addition, the number of owned cats was estimated to be around 322,678 in Umbria and Marche combined (of which at least 124,000 were allowed to roam outdoors), compared to 168,470 from Campania (with 128,650 free roamers). These estimates should be considered as mere approximations, as cat ownership in Italy is not subject to mandatory registration and the data presented are now almost two decades old, meaning that the true numbers are likely to be higher. A serological screening carried out on 304 cats sampled across farms from Southern Italy where livestock tested positive to *T. gondii* antibodies revealed that 98% of cats (298/304) were also seropositive while no one was in the active oocyst shedding phase at the time of sampling [30]. Along with location, age class was identified as a significant risk factor for *T. gondii* infection. This finding was to be expected and is not unprecedented [16,27,31], as it is a direct consequence of the accumulation hypothesis. Since *T. gondii* is a food-borne pathogen, the longer an animal forages, the higher the likelihood of coming in contact with the parasite; thus, older animals have an increased probability of exposure [31]. The results also reflected the global trend observed by Wei et al. [21], which reported higher seroprevalence in females compared to males. However, neither this study nor Wei et al. [21] found this difference to be statistically significant. The absence of a significant association between sex and *T. gondii* seropositivity could be explained by the ecology of red foxes; both males and females are opportunistic feeders that usually occupy overlapping home ranges whilst using the same foraging strategies, leading to comparable exposure levels.

Most of studies focused on *E. cuniculi* in red foxes relied on molecular techniques from various matrices [2,8,32], whereas just one study from the United Kingdom examined the presence of antibodies using a direct agglutination test [33]. This study accounts for the first attempt to use the IFAT to search for *E. cuniculi* in red foxes, detecting a seroprevalence of 15% (18/120). While the detected prevalence is lower than the 45% reported in the UK [33], it still indicates that a notable portion of the fox population has been exposed to the parasite. Serological approaches are particularly valuable in wildlife studies as they allow us to perform large-scale screening and are more sensitive compared to molecular methods that can underestimate true prevalence [34]. Since pooled prevalence of microsporidia in Italy (39.4%) and the United Kingdom (33.9%) is comparable [35], the difference in seropositivity between the Italian and the British fox population could stem from the difference in methodology used for the detection of the parasite. While the ELISA and IFAT are considered the gold standard for the detection of microsporidia [36,37], the lack of specific anti-fox secondary antibodies and the use of anti-dog antibodies might have hindered the detection of positives with very low titers. Seropositivity to *E. cuniculi* was not associated with sex, age class or geographic location in both this study and in the study carried out by Meredith et al. [33]. Nevertheless, the presence of *E. cuniculi* in both Central and Southern Italy reflects a widespread distribution of the parasite and comparable levels of exposure across the longitudinal gradient. Notably, 83.3% (15/18) of *E. cuniculi*-positive foxes were concurrently seropositive for both *T. gondii* and *E. cuniculi*, with statistical analysis revealing a significant association between the two pathogens (OR = 4.9, *p* = 0.03). Such an association has been reported before in only one fox that was seropositive to *T. gondii* and from which *E. cuniculi* DNA was detected [8]. According to these findings, foxes exposed to one parasite might be at risk to come in contact with the associated parasite, potentially due to the overlapping transmission routes like carnivorism or environmental contamination [38].

The low prevalence (3.3%) detected in the present study represents the first serological assessment of *N. caninum* in red foxes from Italy. This result is in line with the seroprevalence reported from other European countries such as Ireland (1.4%, [39]; 3%, [8]), Hungary (1.5%, [24]), the United Kingdom (0.9%, [40]; 2%, [41]) and the Czech Republic (3.8%, [26]). Other studies reported both higher levels of seroprevalence in Belgium (17%, [42]) and no detections in Sweden (0%, [43]). The results obtained in Italy suggest a low level of exposure to *N. caninum* in red foxes from Central and Southern Italy, if compared to the outcomes of vixen in Northwestern Italy by showing a molecular prevalence of 35.3% (6/17) from [44]. This discrepancy in prevalence could be attributed first to the different sample size among the two studies, but also to the different environments from which the foxes were sampled. In fact, cattle farming is more present in Northern Italy [45] where, especially in the alpine regions, cattle are extensively reared in open pastures on mountains and valleys that are usually in direct continuity with the wild environment, therefore, allowing for the intersection of the sylvatic and domestic cycle of *N. caninum*. Vertical transmission has been proved to be an effective transmission route for *N. caninum* in red foxes [44] as for other species, but their epidemiological role in the parasite’s life cycle is yet to be clarified as there is limited evidence on the active shedding of oocysts, and the role of intermediate or dead-end host rather than a definitive host [46,47] is suggested. None of the risk factors have proven to be a significant predictor of infection, meaning that, contrary to *T. gondii*, *N. caninum* does not seem to be influenced by cumulative exposure but rather by occasional events of exposure.

## 5. Conclusions

The present study provides new insights on the seroprevalence of *T. gondii*, *N. caninum* and *E. cuniculi* in red foxes from Italy, reinforcing the value of this wild species as an epidemiological sentinel for the circulation of pathogens of veterinary and medical concern, in both wild and urban environments. The results support the hypothesis of infection risk rising with age due to more opportunities of exposure, and that red foxes are simultaneously exposed to multiple food-borne pathogens, possibly due to their ecological role as scavengers. Overall, these findings highlight the importance of wildlife surveillance to better understand the ecology and circulation of parasites, to inform both public health and livestock management strategies.

## Figures and Tables

**Table 1:** Seroprevalences for *Toxoplasma gondii*, *Neospora caninum* and *Encephalitozoon cuniculi* in red foxes stratified by epidemiological risk factors (sex, age class and location) and status at the time of sampling.

Parasite	Risk Factor	Category	Ratio	Prevalence	95% CI
*T. gondii*	Sex	Male	45/70	64.3%	52.6–74.5
		Female	34/50	68.0%	54.2–79.2
	Age class	Adult	46/58	79.3%	67.2–87.7
		Juvenile	33/62	53.2%	41.0–65.1
	Location	Central Italy	48/81	59.3%	48.4–69.3
		Southern Italy	31/39	79.5%	64.5–89.2
	Status	Alive	33/60	55.0%	42.3–67.2
		Deceased	46/60	76.7%	64.8–86.6
*N. caninum*	Sex	Male	3/70	4.3%	1.5–11.9
		Female	1/50	2.0%	0.4–10.5
	Age class	Adult	3/58	5.2%	1.8–14.1
		Juvenile	1/62	1.6%	0.3–8.6
	Location	Central Italy	3/81	3.7%	1.3–10.3
		Southern Italy	1/39	2.6%	0.5–13.2
	Status	Alive	0/60	0.0%	0.0–0.0
		Deceased	4/60	6.7%	2.2–15.3
*E. cuniculi*	Sex	Male	10/70	14.3%	7.9–24.3
		Female	8/50	16.0%	8.3–28.5
	Age class	Adult	12/58	20.7%	12.3–32.8
		Juvenile	6/62	9.7%	4.5–19.5
	Location	Central Italy	13/81	16.0%	9.6–25.5
		Southern Italy	5/39	12.8%	5.6–26.7
	Status	Alive	8/60	13.3%	6.4–23.7
		Deceased	10/60	16.7%	8.8–27.7

**Table 2:** Outcomes of the multivariable logistic regression with stepwise selection of epidemiological factors used as predictor of *Toxoplasma gondii* infection in red foxes from Central and Southern Italy.

Risk Factor	Category	Seroprevalence	OR	95% CI	*p*
Age class	Juvenile	53.2% (33/62)	0.29	0.12–0.65	0.004
	Adult	79.3% (46/58)	1 (ref.)	-	-
Location	Southern Italy	79.5% (31/39)	2.72	1.11–7.23	0.034
	Central Italy	59.3% (48/81)	1 (ref.)	-	-

## Data Availability

All data generated from this research was disclosed in the article.

## Abbreviations

The following abbreviations are used in this manuscript:

IFAT	Immuno-Fluorescent Antibody Test
ELISA	Enzyme-Linked Immuno-Sorbent Assay
DAT	Direct Agglutination Test
